# African American Food Environments and Anti-Inflammatory Intake in Pregnancy

**DOI:** 10.3390/ijerph23050646

**Published:** 2026-05-13

**Authors:** Najjuwah Walden, Rachel Tabak, Derek S. Brown, Joan Luby, Barbara Warner, Tara Smyser, Cynthia Rogers, Deanna Barch, Sarah K. England, Tonni Oberly, Christopher Smyser, Lora Iannotti

**Affiliations:** 1 Department of Psychiatry, Washington University School of Medicine in St. Louis, St. Louis, MO 63110, USA; 2 School of Public Health, Washington University in St. Louis, St. Louis, MO 63110, USA; 3 Department of Pediatrics, Washington University School of Medicine in St. Louis, St. Louis, MO 63110, USA; 4 Department of Psychological & Brain Sciences, Washington University in St. Louis, St. Louis, MO 63130, USA; 5 Department of Obstetrics & Gynecology, Washington University School of Medicine in St. Louis, St. Louis, MO 63110, USA; 6 College of Public Health, The Ohio State University, Columbus, OH 43210, USA; 7 Department of Neurology, Washington University School of Medicine in St. Louis, St. Louis, MO 63110, USA

**Keywords:** food environment, fast-food, grocery store, convenience store, food intake, diet, prenatal health, urban health

## Abstract

**Highlights:**

**Public health relevance—How does this work relate to a public health issue?**

- This study addresses how dietary intake of nutrient-dense, anti-inflammatory foods among African Americans in pregnancy is explained by spatial determinants.
- It demonstrates that legume intake is independently associated with residential proximity to the nearest grocery store and fast-food restaurant in the St. Louis metro area.

**Public health significance—Why is this work of significance to public health?**

- Although previous research has investigated spatial determinants of prenatal diet quality, no studies have assessed how these factors affect the consumption of specific nutrient-dense foods essential for maternal–fetal health.
- This study underscores the significance of accounting for spatial determinants when assessing food choice and intake frequency in pregnancy.

**Public health implications—What are the key implications or messages for practitioners, policy makers and/or researchers in public health?**

- Given the importance of nutrient-dense, anti-inflammatory foods for maternal–fetal health, future research should assess the extent of food processing in grocery-store and fast-food-restaurant purchases and its impact on maternal–fetal outcomes.
- Further interventions and research are needed to assess the impact of decreasing the distance between African American communities and healthy food environments.

**Abstract:**

Understanding the spatial determinants of food intake is crucial for establishing links between maternal food environments, diet, and health outcomes. Therefore, this study aimed to identify how the distance and density of built food environments in proximity to African American pregnant women living in urban settings are associated with nutrient-dense, anti-inflammatory food intake. We hypothesized that living closer to grocery stores and farther from fast-food restaurants and convenience stores is associated with increased intake. We also hypothesized that higher grocery-store density and a lower density of fast-food restaurants and convenience stores is associated with increased intake. Using cross-sectional data from the Early Life Adversity, Biological Embedding (eLABE) and Risk for Developmental Precursors of Mental Disorders Study, as well as geographic information system (GIS) data and linear regression analyses, we examined the relationships between the built food environment and food intake in the St. Louis metropolitan area, adjusting for covariates. This analysis revealed that shorter distance to fast-food restaurants and longer distance to grocery stores were associated with higher legume intake in adjusted models. These findings highlight nuanced and counterintuitive associations, underscoring the need for additional research to understand why more distant grocery stores and closer fast-food restaurants are linked to higher legume intake among African Americans.

## 1. Introduction

Prior research suggests that across all racial and ethnic groups in the United States, diet quality is lowest among African Americans before conception and in all trimesters of pregnancy [1]. High diet quality is defined as a diversified and balanced diet with anti-inflammatory potential that provides sufficient energy and nutrients to meet the needs of a living being [2,3]. According to the American College of Obstetricians and Gynecologists (ACOG), critical nutrients during pregnancy include omega-3 polyunsaturated fatty acids; calcium; iron; iodine; choline; and vitamins A, C, D, and B (B6, folate, and B12) [4]. Anti-inflammatory foods with high concentrations of these nutrients include dark, leafy green and red–orange vegetables; melon, berry, and citrus fruits; nuts and seeds; legumes; and fish [4,5,6,7,8,9,10,11]. However, prior research suggests that fiber and micronutrient intake is inadequate among African Americans and that most calories within this population come from fat and carbohydrates [12]. In earlier investigations conducted with African American pregnant women living in urban neighborhoods, difficulty accessing food close to home and time constraints that made fast food and processed snacks more convenient could explain food choices high in fat [13]. However, additional research is needed to identify whether the geospatial landscape of predominantly African American urban built food environments can explain dietary intake of nutrient-dense, anti-inflammatory foods.

Urban built food environments vary significantly from rural environments [14,15,16]. In urban neighborhoods, fast-food restaurants exist every 0.73 miles, and having a higher concentration of fast-food restaurants close to home is associated with increased consumption of foods high in calories, carbohydrates, saturated fats, and sodium when comparing urban and rural neighborhoods [17,18,19]. Supermarkets are also fewer in number and farther in distance in urban socioeconomically disadvantaged neighborhoods compared to urban neighborhoods with higher socioeconomic status [20]. As neighborhood poverty increases, there are significantly more convenience stores in predominantly African American impoverished neighborhoods than in predominantly White impoverished neighborhoods [16]. Pregnant women who live farther from grocery stores yet closer to fast-food restaurants and convenience stores are also at a higher risk for intrauterine growth restriction, gestational diabetes, hypertension, preterm birth, and low-birth-weight infants [21,22]. Dietary inflammatory potential appears to influence these outcomes primarily through its impact on oxidative stress pathways, disruption of nutrient homeostasis, alterations in fetal metabolic programming, impairment of endothelial function, and modulation of gut microbial diversity and activity [23,24]. Thus, lower diet quality in urban built food environments is significantly associated with diet-related maternal and infant morbidities [25,26]. However, while research has assessed the relationship between urban built food environments and intake of food with high inflammatory potential among African American pregnant women, research has yet to evaluate how these environments are related to intake of foods with anti-inflammatory potential.

Persistent gaps in nutrient intake among African American pregnant women from preconception through postpartum raise important questions about the structural factors that shape diet quality and food intake. Growing evidence indicates that these patterns reflect the constraints and opportunities created by the built food environment. Therefore, the current study aimed to evaluate the relationship between the built food environment of African American pregnant women living in the St. Louis metropolitan area and their prenatal intake of nutrient-dense, anti-inflammatory foods—dark, leafy green and red–orange vegetables; melon, berry, and citrus fruits; nuts and seeds; legumes; and fish high in omega-3 —which increase diet quality and supply ACOG-recommended nutrients for healthy pregnancy [4,5,6,7,8,9,10,11]. Nutrient-dense, anti-inflammatory foods are crucial for reducing the risk of perinatal depression and anxiety, miscarriage, pre-eclampsia, hemorrhage, low birth weight, preterm delivery, and fetal abnormalities due their effects on various physiological processes [27,28,29]. Therefore, this study aims to identify whether residential proximity to fast-food restaurants, convenience stores, or grocery stores is a determinant of intake of nutrient-dense, anti-inflammatory foods needed to support healthy pregnancy outcomes.

## 2. Materials and Methods

### 2.1. Study Design and Setting

The sample included participants of the Early Life Adversity, Biological Embedding (eLABE) and Risk for Developmental Precursors of Mental Disorders Study at Washington University School of Medicine in St. Louis. The multi-wave, longitudinal eLABE study conducted in partnership with the March of Dimes Prematurity Research Center at Washington University School of Medicine in St. Louis aimed to investigate how experiences in pregnancy affect child development and the well-being of mothers and caregivers. The eLABE study is briefly described here; additional details about the study are available elsewhere [30,31].

Participants were recruited from two OBGYN clinics by March of Dimes. One clinic primarily serves patients without insurance and Medicaid/Medicare insurance. The other clinic primarily serves a patient population with private health insurance. Patients without known pregnancy complications, fetal congenital abnormalities, or substance use during pregnancy (excluding tobacco and marijuana) were invited to participate in the eLABE study. The study recruited 395 pregnant women from 2017 to 2020. Inclusion criteria stipulated English-speaking participants and a maternal age 18 years or older. The study did not exclude non-U.S. citizens. The Washington University School of Medicine Institutional Review Board granted ethics approval ahead of study activities, and all participants provided written informed consent. A sample of 395 provides 80% power to detect the effects of distance to the nearest fast-food restaurant and convenience store on food intake ($f^{2}$ = 0.03), consistent with prior research [32].

This analysis aimed to evaluate the relationship between the distance and density of built food environments and dietary intake of nutrient-dense, anti-inflammatory foods among African American pregnant participants, controlling for covariates previously evidenced to be associated with prenatal diet and nutrient intake, including infant sex, perceived stress, maternal age, insurance coverage, employment status, marital status, maternal education, income-to-needs ratio, the Area Deprivation Index, body mass index (BMI), diabetes, parity, prior miscarriage, and substance use [33,34,35,36,37,38,39,40,41,42,43,44,45,46,47]. Of those enrolled in the eLABE study, 87 (35% of African American participants) met the inclusion criteria for the current analysis, which were: African American racial identity and providing complete residential address data, including street address, city, state, and zip code, at the time of eLABE study enrollment. Additional inclusion criteria include complete National Institutes of Health (NIH) National Cancer Institute (NCI) Diet History Questionnaire II (DHQ-II) data, completed online at 21 to 40 weeks gestational age.

### 2.2. Variables

#### 2.2.1. Dietary Intake

The NIH NCI DHQ-II was administered in the third trimester to assess the portion size and frequency of 134 food items consumed over the past 12 months. Frequency was assessed by asking how often, on average, participants consumed food items, with responses including 1 time per month or less, 2–3 times per month, 1–2 times per week, 3–4 times per week, 5–6 times per week, 1 time per day, 2–3 times per day, 4–5 times per day, and 6 or more times per day [48]. Frequency and quantity were multiplied to determine the average daily intake for each food item using the Diet*Calc Analysis Program, Version 1.5.0 for DHQ-II (National Cancer Institute, Bethesda, MD, USA) [49]. This study aims to evaluate intake of dark, leafy green and red–orange vegetables; melon, berry, and citrus fruits; nuts and seeds; legumes; and fish high in omega-3 due to the high availability of omega-3 polyunsaturated fatty acids; calcium; iron; iodine; choline; and vitamins A, C, D, and B (B6, folate, and B12) essential for healthy fetal development and prenatal health in these anti-inflammatory foods [4,5,6,7,8,9,10,11]. The DHQ-II aggregates the dietary intake of citrus, berry, and melon fruits (oranges, tangerines, clementines, grapefruit, strawberries, cantaloupe, watermelon, and honeydew); dark, leafy green vegetables (broccoli, romaine, and spinach); red–orange vegetables (bell pepper, carrot, pumpkin, sweet potato, and tomato); legumes (baked beans, pinto beans, kidney beans, refried beans, snow peas, blackeye peas, lima beans, lentils, and soybeans); nuts and seeds (peanuts, walnuts, almonds, flaxseeds, sunflower seeds, and pumpkin seeds); and fish high in omega-3 (salmon, tuna, and trout). This study measured intake of these food groups as continuous outcome variables.

#### 2.2.2. Built Food Environment

Downs et al. (2020) define the built food environment as the retail food environment [50]. This analysis focused on the built food environment within a certain distance of a participant’s home where people usually engage in the retail sale of canned, dry, fresh, and prepared foods [51,52]. These included supermarkets and grocery stores, convenience stores, and restaurants [51]. This study defined a supermarket as a large chain grocery store with a supermarket Standard Industrial Code (SIC), such as Walmart [50,52]; a grocery store as a local, regional, or national chain with a grocery-store SIC [50,52]; a convenience store as a food mart without a gas station with a convenience-store SIC [52]; and a restaurant as a location where prepared meals are sold for sit-down service, take-out, or delivery with a restaurant SIC [50]. This study limited its analysis of restaurants to fast-food vendors, defined as locations where processed food is served as a quick meal or takeaway, to test the hypothesis that fast-food proximity is associated with food choice and intake frequency [53].

This study obtained a comprehensive list of 4155 names and street addresses of Missouri and Illinois retail food vendors from the 2018–2020 ArcGIS Business Analyst Pro (Esri, Redlands, CA, USA) directories to case-match locations in proximity to participants on the DHQ-II completion date. A total of 3300 retail food vendors were omitted due to duplication of entries, incomplete street addresses, non-food retail sales, or retail food (i.e., convenience-store and fast-food) sales at gas stations. Gas-station convenience stores were omitted to test the hypothesis that stand-alone convenience-store proximity is associated with food choice and intake frequency, as demonstrated by Zenk et al. (2012) with non-pregnant African American adults [32,54]. This study also removed wholesale distributors, record labels, banquet halls, hotel retailers, and corporate office retailers with convenience-store or restaurant SICs from the analysis. Food stores were then categorized into three groups—grocery stores (including supermarkets), convenience stores, and fast-food restaurants—for a total of 855 observations.

**Figure A1:**
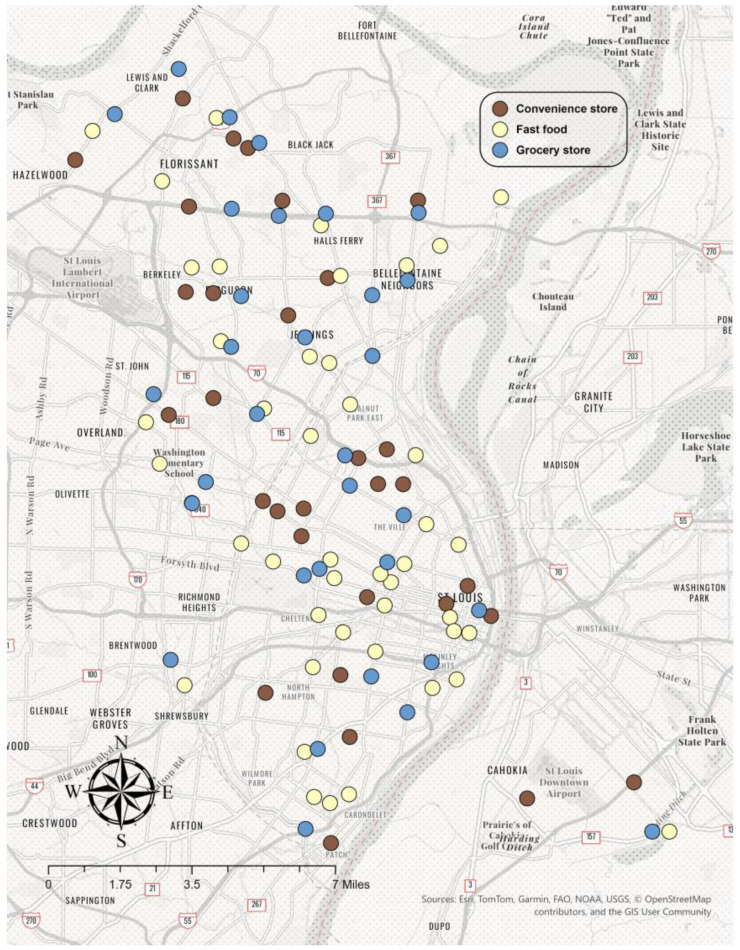
Map of food vendors in proximity to residential addresses of 87 African American pregnant women in the St. Louis Metropolitan Area, 2018–2020.

ArcGIS StreetMap Premium (ArcGIS Pro 3.1 Patch 7, Esri, Redlands, CA, USA) was used to calculate network distance and density measurements. Network distance was defined as the length of the shortest path walking away from the participant’s residential address towards the specified food vendor (e.g., convenience store, fast-food restaurant, or grocery store) along streets and measured as a continuous variable. The network distance between each participant’s residence and each convenience store, fast-food restaurant, and grocery store was calculated to identify the distance to the closest vendor. Density was defined as a count of specified food vendors within a 0.5-mile network service area of each participant’s residence and measured as a discrete variable. This study calculated the quantity of convenience stores, fast-food restaurants, and grocery stores that were in the service area of each participant’s residential address. Both network distance and the 0.5-mile buffer were defined along walkable streets (excluding highways). A total of 33 convenience stores, 52 fast-food restaurants, and 35 grocery stores were in proximity to participants included in the analysis (see Figure A1 and Table A1).

#### 2.2.3. Covariates

The eLABE study collected self-reported baseline data on participant date of birth, residential address, household income, household size, and highest level of education. Self-administered surveys also measured perceived stress, alcohol use, and cigarette smoking (including e-cigarettes) prenatally. Marital status, employment status, insurance coverage, maternal pre-pregnancy BMI, infant sex, prior live birth, prior miscarriage, and diabetes status were abstracted from patient electronic health records at labor and delivery for the eLABE study and used for analyses. Infant sex was described as boy or girl. Perceived stress is an ordinal covariate measured using Cohen’s Perceived Stress Scale (PSS-10) [55,56]. PSS-10 scores scale from 0 to 40, indicating low stress (0–13), moderate stress (14–26), and high stress (27–40) [55]. The income-to-needs ratio (INR) is a continuous covariate measured by dividing the household income by the federal poverty level corresponding to the family size for the specific year of data collection [57,58]. Residential addresses were geocoded by the eLABE study, assigning latitude and longitude coordinates and census block designations to measure the national Area Deprivation Index (ADI), an ordinal measure, with scores closer to 100 signifying higher levels of deprivation [59]. Diabetes was categorized as the presence of pregestational or gestational diabetes (yes) or the absence of both (no) based on chart review. Pregestational diabetes is defined as diabetes mellitus diagnosed before the pregnancy begins, while gestational diabetes is hyperglycemia that develops during pregnancy and resolves after labor and delivery [60,61]. This study measured pre-pregnancy BMI reported from patient EHRs as a continuous covariate. BMI scores range from 12 to 65, indicating underweight (<18.5 kg/$m^{2}$), normal weight (18.5–24.9 kg/$m^{2}$), overweight (25.0–29.9 kg/$m^{2}$), class I obesity (30.0–34.9 kg/$m^{2}$), class II obesity (35.0–39.9 kg/$m^{2}$), and class III/morbid obesity (≥40 kg/$m^{2}$) [62,63].

### 2.3. Statistical Analyses

**Figure 1:**
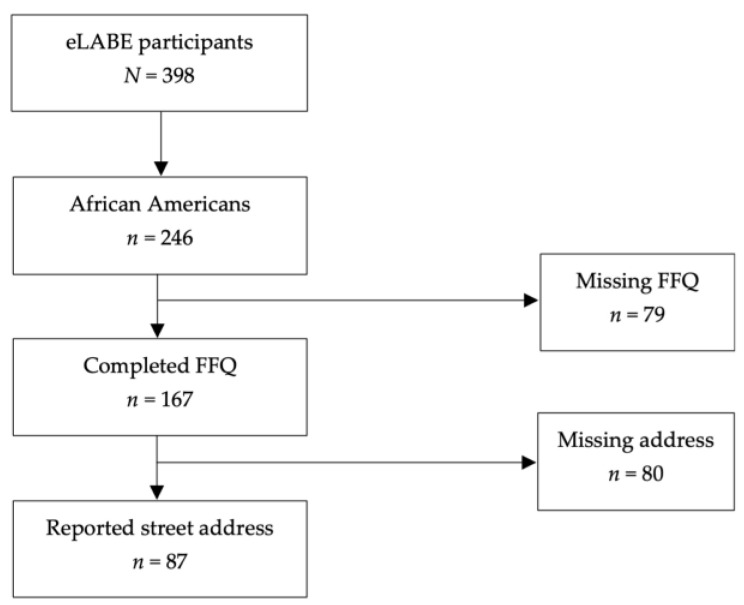
Flow diagram of participant inclusion and exclusion criteria.

The data were initially screened for missing values in the primary exposure and outcome variables (Figure 1). African American participants with missing primary exposure and outcome variable data were excluded from the analysis (*n* = 158, 65% of African American participants). Covariates with missing data included education level, employment status, area deprivation, perceived stress, pre-pregnancy BMI, prior miscarriage, diabetes diagnosis, and cigarette use. The Shapiro–Wilk test assessed the normality of each variable. Because the primary outcomes were not normally distributed, descriptive statistics were reported as median, minimum, and maximum values, and bivariate analyses were conducted using Kruskal–Wallis, Spearman, and Mann–Whitney tests. Kruskal–Wallis tests assessed associations between dietary intake and categorical variables with more than two levels (i.e., employment status and maternal education), while Mann–Whitney tests were used for categorical variables with two levels (i.e., marital status, insurance coverage, parity, miscarriage history, diabetes status, infant sex, alcohol use, and cigarette smoking). Spearman’s rank correlation examined associations between dietary intake and continuous covariates (i.e., maternal age, INR, ADI, pre-pregnancy BMI, and perceived stress). In addition, Spearman tests were used to assess associations between dietary intake and built food environment distance, while Kruskal–Wallis tests were used for built food environment density. Associations between built food environment density and covariates were examined using Pearson’s chi-square test for categorical variables and Kruskal–Wallis tests for continuous variables. Significant associations were confirmed using Dunn–Bonferroni post hoc tests, and potential confounders of dietary intake and built food environment measures were included in adjusted multivariate models.

Multivariate linear regressions were conducted to estimate the conditional association of distance and density measures of the built food environment with the average daily prenatal intake of melon, berry, and citrus fruits; dark, leafy green and red–orange vegetables; nuts and seeds; legumes; and fish high in omega-3 consumed in the last 12 months, adjusted for potential confounders. Prior to conducting multivariate analyses, this study tested for collinearity among confounders and removed variables with a correlation coefficient >0.7. Given the non-normal distribution of dietary intake and heterogeneity of variances, Box–Cox transformations were applied to multivariate regressions to approximate a normal distribution for the outcome variables. This transformation was executed for each dietary intake model before performing each regression. This study includes multivariate regression tables, with dietary outcomes significantly associated with distance and density measures of the built food environment in univariate analyses. The additional models are included in Supplementary Materials. All statistical analyses were conducted using Stata 18 (StataCorp LLC, College Station, TX, USA).

## 3. Results

### 3.1. Study Population

The analysis includes 87 African American pregnant women over 18 years of age (Table 1). Compared to all participants in the eLABE study (*n* = 398), this analysis includes women with less education, and fewer were employed, married, and covered by private insurance. Pregnant women living in areas with the least area deprivation are also not represented in the analysis. The highest level of education was college graduation, and 60.3% were employed. The sample includes women who were single (86.2%), married (12.6%), or divorced (1.1%). Women were insured by Medicaid/Medicare (50.6%) or private insurance (33.3%) or were uninsured (16.1%). The sample lived at or below the federal poverty level (52.6%), above but below two-fold the federal poverty level (38.2%), or above two-fold the federal poverty level (9.2%). Women lived in areas within each quintile of the Area Deprivation Index: low (20.9%), below average (22.1%), average (19.8%), above average (18.6%), and high deprivation (18.6%). The sample reported low (44%), moderate (50.7%), and high (5.3%) psychosocial stress in the third trimester. Most were pregnant with boys (60.9%) and multiparous (60.9%). Pre-pregnancy BMI ranged from underweight (<18.5 kg/m^2^) to morbidly obese (≥40 kg/m^2^) (Table 1). Pregestational or gestational diabetes diagnosis was reported in 4.6% of the sample. Prior miscarriage was reported by 30.6%. Alcohol and cigarette use was reported by 5.75% and 10.59% of women, respectively.

The shortest distance to the nearest convenience store, fast-food restaurant, and grocery store was 0.1 miles, 0.2 miles, and 0.5 miles, respectively. The maximum number of convenience stores, fast-food restaurants, and grocery stores within a 0.5-mile radius of the participants’ residence was 3, 3, and 2, respectively. Median daily intake in the last 12 months was 1.2 cups of melon, berry, and citrus fruits (IQR: 0.4–2.2 cups); 0.3 cups of dark, leafy green and red–orange vegetables (IQR: 0.2–0.8 cups); 0.2 ounces of nuts and seeds (IQR: 0.1–0.7 oz.); 0.1 cups of legumes (IQR: 0.0–0.3 cups); and 0.0 ounces of fish high in omega-3 (IQR: 0.0–0.0 oz.).

### 3.2. Built Food Environments and Associated Food Intake

In bivariate analyses, tests indicated no statistically significant differences in the intake of select foods in association with the distance or density of convenience stores or grocery stores in proximity to the participants’ residential addresses (Table 2). In addition, fast-food density was not statistically significantly associated with select food intake. However, results revealed a statistically significant negative association between legume intake and distance between a participant’s home and the nearest fast-food restaurant (ρ = −0.24, *p* = 0.03), suggesting legume intake is higher among participants living closer to fast-food restaurants (Table 2). After applying a Bonferroni correction for multiple comparisons, the relationship remained statistically significant (*p* < 0.05). This study did not detect statistically significant associations between the distance or density of food environments and the intake of melon, berry, and citrus fruits; dark, leafy green and red–orange vegetables; nuts and seeds; or fish high in omega-3 (Table 2).

### 3.3. Covariates of Built Food Environments and Intake

Bivariate tests indicated built food environment distance and density were significantly associated with infant sex, marital status, INR, ADI, and alcohol use. However, after Bonferroni correction for multiple comparisons, only the relationships between convenience-store distance and infant sex (*p* < 0.05) and grocery store density and alcohol use (*p* < 0.05) remained statistically significant. Dietary intake was also significantly associated with maternal age at delivery, parity, miscarriage history, and alcohol use in bivariate analyses. However, after Bonferroni correction for multiple comparisons, no associations met the adjusted significance threshold (*p* < 0.05). Therefore, only infant sex and alcohol use were included in multivariate models to control for the effects of potential confounders.

### 3.4. Multivariate Models of Food Intake Predicted by the Built Food Environment

The multivariate linear regression models assessed the association between distance and density measures of the built food environment and select food intake, with adjusted models controlling for potential confounders (infant sex and alcohol use). The results of legume intake without potential confounders (Model 1) revealed that fast-food distance, grocery-store distance, and grocery-store density were statistically significantly associated with legume intake (Table 3). However, after Bonferroni correction for multiple comparisons, only fast-food distance and grocery-store distance remained statistically significant. As the distance to fast-food restaurants decreases, legume intake increases (β = −0.04, *p* = 0.01), while further distance to grocery stores is associated with higher legume intake (β = 0.04, *p* = 0.01). The overall F-test was statistically significant (F (6, 80) = 2.35, *p* < 0.05).

After adjusting the multivariate regression models and including potential confounders (Model 2), closer distance to fast-food restaurants (β = −0.05, *p* = 0.00) and further distance to grocery stores (β = 0.04, *p* = 0.01) remained statistically significantly associated with higher legume intake (Table 3). The overall F-test was marginally significant (*p* = 0.06). Additionally, there were no significant relationships between potential confounders and dietary intake in the adjusted models.

This study did not detect significant associations between the distance and density of convenience stores, fast-food restaurants, or grocery stores and the intake of melon, berry, and citrus fruits; dark, leafy green and red–orange vegetables; nuts and seeds; or fish high in omega-3 in multivariate models (Supplemental Material Table S1).

## 4. Discussion

This study aimed to evaluate the relationship between the distance and density of convenience stores, fast-food restaurants, and grocery stores in proximity to African American pregnant women living in urban settings and the dietary intake of nutrient-dense, anti-inflammatory foods recommended by ACOG. Based on previous research, this study hypothesized that living closer to grocery stores and farther from fast-food restaurants and convenience stores is associated with increased prenatal intake of nutrient dense, anti-inflammatory foods. This study also hypothesized that a higher density of grocery stores and a lower density of fast-food restaurants and convenience stores is associated with increased nutrient-dense, anti-inflammatory food intake. In contrast to the hypotheses, results indicated a significant association between legume intake and the presence of grocery stores and fast-food restaurants among African American pregnant women living in the St. Louis metropolitan area in the opposite direction of what was expected. Legume intake increased as distance to the nearest fast-food restaurant decreased and distance to the nearest grocery store increased.

### 4.1. Distance to Nearest Grocery Store

This study did not expect that greater distance to the nearest grocery store would be associated with higher intake. This runs counter to the hypothesis that closer proximity to grocery stores is associated with higher dietary intake; however, the results are similar to previous findings that supermarkets with higher nutritional scores are farther from neighborhoods with higher percentages of African American populations and that these stores are sought out to increase healthy food intake [20,21]. While the accessibility of Aldi, Save-A-Lot, Schnucks, and Shop ‘N Save was geographically dispersed in the study, locations that are farther from African American neighborhoods may have greater availability of nutrient-dense foods. Alternatively, households living farther from grocery stores may adapt by planning infrequent shopping trips and purchasing shelf-stable foods such as dried or canned legumes [64]. Higher quantities of shelf-stable foods within the household at lower price points, compared to perishables, may also explain why the models were powered to explain legume intake [65]. Additionally, food purchasing decisions are frequently shared among social networks and the distance to the nearest grocery store may not reflect the distance that participants have to travel [66]. The food purchasing behaviors of partners, parents, siblings, and friends may help explain legume consumption. Taken together, these findings highlight the importance of considering both structural access and household strategies in food environment research.

### 4.2. Distance to Nearest Fast-Food Restaurant

The study also identified an unexpected finding describing the relationship between fast-food restaurant distance and legume intake. Prior literature often links greater fast-food availability with poorer diet quality [67,68,69], lower legume intake [70], or no relationship to legumes grouped into vegetables [71]. However, to the knowledge of the authors, the current study is the first to identify an inverse relationship. Living closer to fast-food restaurants was associated with higher legume intake. One possible explanation is that fast-food restaurants in the analysis—including Taco Bell, Popeyes Louisiana Kitchen, Wendy’s, Lee’s Famous Recipe Chicken, Church’s Chicken, and KFC—offer menu items containing legumes such as pinto beans, red kidney beans, baked beans, and green beans. This may suggest that living closer to these vendors increases opportunities to consume legumes in these forms. Alternatively, the results may suggest legumes are more likely to be consumed if pre-prepared. Purchasing pre-prepared canned or restaurant-sourced legumes significantly reduces cooking time and effort barriers of consumption [72,73]. Therefore, increasing the availability of pre-prepared legumes may increase legume consumption. However, it is important that populations do not rely on fast-food restaurants for legume consumption for “anti-inflammatory” intake.

Fast-food restaurants commonly sell ultra-processed foods [74], challenging the assumption that legume intake from these locations is inherently anti-inflammatory when they are ultra-processed rather than minimally processed. Minimally processed foods are whole foods altered only by the removal of inedible or unwanted parts and preparation methods that maintain a higher level of nutrients [75,76], whereas ultra-processed foods are chemically modified formulations that promote low-grade inflammation [75,77]. While unprocessed or minimally processed legumes are nutrient-dense and anti-inflammatory, promoting digestion and absorption of amino acids highly recommended for infants, ultra-processed methods are linked to risk of higher gestational weight gain, gestational diabetes, lower levels of essential nutrients in breastmilk, and higher odds of offspring obesity [78,79]. Although the findings suggest that living closer to fast-food restaurants is associated with higher legume intake, further research is needed to determine whether this intake is derived from minimally processed or ultra-processed legumes and acceptable anti-inflammatory replacements.

### 4.3. Legumes

Black-eyed peas, broad beans, butter beans, chickpeas, cowpeas, kidney beans, lentils, lima beans, and pigeon peas are central to African American food choices [80]. African American food choices have been generationally shaped by continental Africa, the American South, the Caribbean, and South America, consisting of many fruits, vegetables, and legumes [80]. Legumes are a valuable source of protein, fiber, iron, calcium, zinc, and folate—nutrients critical to maternal and fetal health [81,82,83,84]. Higher legume consumption has also been associated with reduced risk of gestational diabetes and low-birth-weight infants in studies of pregnant women in Spain and Iran [85,86]. Therefore, the current findings may point to important opportunities to improve maternal and infant health among African Americans by increasing intake of anti-inflammatory legumes. However, while increasing legume intake is promising, intervention studies are sparse, and no prior study has evaluated how to change legume intake from ultra-processed and highly inflammatory to minimally processed and anti-inflammatory.

Future research should examine how to increase legume intake alternatives to legumes consumed from fast-food restaurants and maternal and infant outcomes of intake among African American pregnant women. It is also important to comparatively investigate how uptake and clinical outcomes vary when legumes are pre-prepared (heat and eat) and unprepared (prep and eat) to identify how preparation methods influence legume uptake and nutrition intake. Additionally, research is needed to identify whether sustaining anti-inflammatory legume intake is associated with improved African American maternal and infant health outcomes. However, dietary adherence cannot be separated from built food environments. Living farther from grocery stores with higher nutritional scores is a barrier to overcome and will continue to shape intake if unaddressed [87].

### 4.4. Overlapping Influences

Although grocery-store distance was not significantly associated with legume intake in the bivariate analyses, this variable emerged as a significant predictor in the multivariate models confirmed using the Dunn–Bonferroni post hoc test. This pattern indicates that the relationship between grocery-store distance and legume intake may be suppressed when considered in isolation but becomes apparent after accounting for environmental and sociodemographic variables. Grocery-store distance may function as an independent predictor of legume intake whose effects are more visible after controlling for potential confounders and overlapping influences. For example, the presence of nearby fast-food restaurants or variation in neighborhood characteristics may suppress the direct association between grocery-store access and dietary intake in bivariate analyses. Once these factors are jointly modeled, the independent contribution of grocery-store distance becomes evident. This underscores the importance of examining built food environment influences within a multivariate framework, since unadjusted associations may underestimate their role in shaping dietary behaviors.

### 4.5. Limitations

As a cross-sectional study with no exogenous variables explaining why foods are sourced or where they are sourced, the analysis cannot establish temporal or causal relationships between the built food environment and dietary intake. The NIH NCI DHQ-II captures food intake over past year but does not link specific foods to purchase location, including meal and grocery delivery applications or telephone orders, and may underestimate or overestimate the influence of food vendors. The eLABE study also did not ask participants how often they ate at restaurants or shopped at grocery stores. Future studies that track purchase behaviors using ecological momentary assessments, food journals, or 24-h dietary recalls could provide greater insight into how and where foods are purchased and how they are processed and allow for an assessment of nutrient density. Capturing day-to-day variation would also allow researchers to evaluate occasions when anti-inflammatory food intake meets the Recommended Dietary Allowance (RDA) for nutrient intake and when it fluctuates, providing a more nuanced understanding of dietary patterns and nutrient adequacy during pregnancy.

The current study did not identify significant relationships between built food environment distance and density and fruit, vegetable, nut and seed, or fish intake. However, this may be explained by the overall low consumption of these foods within the sample. Most participants did not report any fish intake, and there was limited variation in the other food groups. This may be due to historical messaging on fish intake during pregnancy, as the FDA and EPA advised limiting fish consumption during pregnancy to minimize methylmercury consumption in 2004 but later recommended two to three servings of fish per week for infant growth and development as of 2017 [88]. However, limited variation in high-omega-3 fish intake may also be due to the sample size (*n* = 87). Additional research with larger sample sizes is needed to detect whether built food environment distance and density explain food intake when there is greater variation in intake or if these relationships remain non-significant. A limitation of this study is the sample size (*n* = 87) of participants who met the inclusion critera. Therefore, while the results provide insight into the relationship between the built food environment and food intake among African American pregnant women in St. Louis, additional research in other urban geographies is needed to identiy the reproducability of these results.

This study also focused on grocery stores, fast-food restaurants, and convenience stores but did not include an exhaustive list of vendors such as gas-station convenience stores and gas-station restaurants. Including these vendors in future analyses may alter or refine the independent associations observed between grocery stores, fast-food restaurants, and legume intake. Furthermore, while geospatial analyses evaluated the distance to the nearest vendor and network density along walkable streets, the eLABE study did not evaluate participants’ primary transportation mode, limiting the ability of the current study to control for the potential confounding effects of transportation, nor did the study validate that the GIS definition of walkable coincides with participant perceptions. Therefore, it is important to evaluate whether the results are replicable when considering travel distance using alternative modes of transportation and considering participant perspectives and perceptions regarding distances and walkability.

It is also important to note that the personal food environment includes accessibility, or the physical distance, time, space and place, individual activity, daily mobility, and mode of transport required to acquire and consume food; desirability, or the preferences, acceptability, tastes, desires, attitudes, culture, knowledge, and skills associated with food choices; convenience, or the relative time and effort of preparing, cooking, and consuming food products; and affordability, or purchasing power, defined as the money and credit available to an individual or a household for spending and consumption of food products [89,90]. Individual factors of the food environment defined by Downs et al. (2020) also include income, values, beliefs, preferences, knowledge, skills, mobility, and time, in addition to social capital and health [50]. The current study measured accessibility—but only in the context of the distance between the built retail food environment and residences. Therefore, this study did not comprehensively control for all measures of the food environment that may influence intake. Future research should study additional food environment determinants to establish their effects on food intake.

## 5. Conclusions

The current study identified that the distance of grocery stores and fast-food restaurants in proximity to African American pregnant women living in the St. Louis metropolitan area is associated with legume intake. Notably, legume intake increased both as the distance to the nearest grocery store increased and as the distance to the nearest fast-food restaurant decreased. These findings highlight that legume consumption is related to the spatial arrangement of food vendors in proximity to African Americans living in urban communities. Importantly, this suggests that even in environments with structural limitations, households may employ strategies that support the intake of nutrient-rich foods such as legumes. However, future research is needed to examine variation in prenatal and fetal outcomes by food processing level, alongside the roles of nutrient intake. Overall, this study underscores the potential to leverage urban built food environments to promote the consumption of foods important for maternal and fetal health by promoting legumes and decreasing the distance between where high-quality foods are located and where African Americans live.

## Figures and Tables

**Table 1:** Characteristics of 87 African American pregnant women in the St. Louis metro area.

Characteristic	Median [Min/Max]/ Number (%)
Maternal age (years)	27.2 [19.1, 41.5]
Maternal education	
Less than high school	7 (10.5%)
High school graduate	52 (77.6%)
College graduate	8 (11.9%)
Employment status	
Unemployed	26 (31.3%)
Student	7 (8.4%)
Employed	50 (60.3%)
Marital status	
Single/Divorced	76 (87.4%)
Married	11 (12.6%)
Insurance status	
Non-Private	58 (66.7%)
Private	29 (33.3%)
Income-to-needs ratio (INR)	0.9 [0.4, 11.8]
Area Deprivation Index (ADI)	86 [31, 100]
Perceived Stress Score (PSS-10)	14.5 [0, 33]
Infant sex	
Boy	53 (60.9%)
Girl	34 (39.1%)
Pre-pregnancy BMI	29.9 [18.1, 64.6]
Prior live birth	
No	27 (31.0%)
Yes	60 (69.0%)
Prior miscarriage	
No	53 (65.4%)
Yes	28 (30.6%)
Pregestational or gestational diabetes	
No	83 (95.4%)
Yes	4 (4.6%)
Alcohol use	
None	82 (94.2%)
Any	5 (5.8%)
Cigarette use	
None	76 (89.4%)
Any	9 (10.6%)
Average daily intake of melon, berry, and citrus fruits (c.)	1.2 [0.0, 24.7]
Average daily intake of dark, leafy green and red–orange vegetables (c.)	0.3 [0.0, 5.0]
Average daily intake of nuts and seeds (oz)	0.2 [0.0, 8.2]
Average daily intake of beans and legumes (c.)	0.1 [0.0, 5.6]
Average daily intake of fish high in omega-3 (oz)	0.0 [0.0, 2.3]
Distance to nearest convenience store	1.9 mi [0.1–4.1]
Distance to nearest fast-food restaurant	1.3 mi [0.2–5.7]
Distance to nearest grocery store	1.8 mi [0.5–5.7]
Density of convenience stores	0 [0, 3]
Density of fast-food restaurants	0 [0, 3]
Density of grocery stores	0 [0, 2]

**Table 2:** Associations between built food environment proximity and average daily prenatal nutrient-dense, anti-inflammatory food intake (*n* = 87) ^1^.

	1. *		2. *		3. *		4. *		5. *	
Built Food Environment Proximity	*Coeff*	*p*-Value	*Coeff*	*p*-Value	*Coeff*	*p*-Value	*Coeff*	*p*-Value	*Coeff*	*p*-Value
Distance to nearest convenience store	0.08	0.45	−0.07	0.51	0.08	0.48	−0.02	0.83	0.06	0.61
Distance to nearest fast-food restaurant	0.04	0.74	−0.09	0.39	−0.08	0.45	−0.24	<0.05	−0.14	0.20
Distance to nearest grocery store	−0.08	0.48	0.02	0.82	−0.06	0.59	0.06	0.58	−0.04	0.69
Density of convenience stores	0.09	0.09	0.43	0.93	0.02	0.99	2.36	0.50	1.50	0.68
Density of fast-food restaurants	4.31	0.23	7.25	0.06	2.07	0.56	7.56	0.06	2.46	0.48
Density of grocery stores	0.14	0.93	1.04	0.60	1.43	0.49	1.77	0.41	2.96	0.23

^1^ Built food environment distance outcomes are reported using Spearman rank correlation estimates, while built food environment density outcomes are reported using Kruskal–Wallis H-test estimates. * 1. melon, berry, and citrus fruits; 2. dark, leafy green and red–orange vegetables; 3. nuts and seeds; 4. Legumes; 5. fish high in omega-3.

**Table 3:** Multivariate linear regression models evaluating the association between built food environment proximity and average daily prenatal legume intake, adjusting for potential confounders (*n* = 87) ^1^.

	Model 1		Model 2	
Variable	β (95% CI)	*p*-Value	β (95% CI)	*p*-Value
Distance to nearest convenience store	0.00 [−0.02, 0.02]	0.99	0.00 [−0.02, 0.02]	0.95
Distance to nearest fast-food restaurant	−0.04 [−0.07, −0.02]	<0.01	−0.05 [−0.08, −0.02]	<0.001
Distance to nearest grocery store	0.04 [0.01, 0.06]	<0.05	0.04 [0.01, 0.07]	<0.01
Density of convenience stores	−0.01 [−0.04, 0.02]	0.60	−0.01 [−0.04, 0.03]	0.70
Density of fast-food restaurants	−0.01 [−0.04, 0.01]	0.36	−0.02 [−0.04, 0.01]	0.26
Density of grocery stores	0.06 [0.01, 0.12]	0.02	0.06 [0.01, 0.13]	0.03 ^†^
Infant sex	-	-	−0.01 [−0.05, 0.02]	0.42
Alcohol use	-	-	0.04 [−0.03, 0.11]	0.28

^1^ β = beta coefficient; CI = confidence interval. Significant coefficients and *p* trend values are in bold print. Model 1: F (6, 80) = 2.35, *p* = 0.038, R^2^ = 0.15. Model 2: F (8, 76.1) = 1.97, *p* = 0.062. ^†^ Type I error, significance attributable to multiple-comparisons problem.

## Data Availability

The original contributions presented in the study are included in the article. Further inquiries can be directed to the corresponding authors.

## Supplementary Materials

The following supporting information can be downloaded at: https://www.mdpi.com/article/10.3390/ijerph23050646/s1, Table S1: Multivariate linear regression models evaluating the association between built food environment proximity and average daily prenatal melon, berry, and citrus fruit intake; Table S2: Multivariate linear regression models evaluating the association between built food environment proximity and average daily prenatal dark, leafy green and red–orange vegetable intake; Table S3: Multivariate linear regression models evaluating the association between built food environment proximity and average daily prenatal nut and seed intake, Table S4: Multivariate linear regression models evaluating the association between built food environment proximity and average daily prenatal intake of fish high in omega-3.

## Abbreviations

The following abbreviations are used in this manuscript:

ACOG	American College of Obstetricians and Gynecologists
ADI	Area Deprivation Index
BMI	Body Mass Index
DHQ-II	Diet History Questionnaire II
EHR	Electronic health record
eLABE	Early Life Adversity, Biological Embedding and Risk for Developmental Precursors of Mental Disorders Study
GIS	Geographic information system
INR	Income-to-needs ratio
kg/m^2^	Kilograms per square meter, a unit of surface density used to measure weight
NCI	National Cancer Institute
NIH	National Institutes of Health
OBGYN	Obstetrics and Gynecology
PSS-10	Perceived Stress Scale
SIC	Standard Industrial Code

## Appendix A

**Table A1:** Names of food vendors in proximity to residential addresses of 87 African American pregnant women in the St. Louis Metropolitan Area, 2018–2020.

Vendor Type	Vendor Names
Convenience Stores	7-Eleven, A&Z Laclede Market, A1 Quick Shop, Arch Quick Mart, BI-LO Market and Package Liquors, Carrie’s Corner Market, DJ’s Mini Mart, Dot’s Neighborhood Place, Express Lande Market, Ferguson Supermarket Inc., Florissant Dairy, Florissant Food & Package Liquor, Florissant Kwik Shop, Grant Mart, In & Out Market LLC, Jarmon’s Convenience Store, Jennings Convenience Center, John’s Market, Kenny’s Discount Cigarettes, Michigan Market, Mini Market, Mitchell’s Express, Omars Food Shop, Quick Stop Food Shop Inc., R&R Mart & Liquor, Regal Food Market, Smart Mart, Unique Market, Village Too, Whistle Stop, Yeatman Market
Fast-Food Restaurants	Arby’s, Burger King, Captain D’s Seafood, Church’s Chicken, DQ Grill & Chill, Jack In The Box, KFC, Lee’s Famous Recipe Chicken, McDonald’s, Popeyes Louisiana Kitchen, Rally’s Hamburgers, Steak ‘N Shake, Subway, Taco Bell, Wendy’s, White Castle
Grocery Stores	Aldi, Save-A-Lot, Schnucks, Shop ‘N Save
