# Supplementary material for: African American Food Environments and Anti-Inflammatory Intake in Pregnancy
Source: Int J Environ Res Public Health. 2026 May 13;23(5):646. doi: 10.3390/ijerph23050646 (PMC13206721; doi:10.3390/ijerph23050646)
Supplement: Supplementary file 1 [file ijerph-23-00646-s001.zip › ijerph-4103572-supplementary.pdf]

Table S1. Multivariate linear regression models predicting average daily prenatal melon, berry, and citrus fruit intake in association with built food environment proximity and potential confounders.*

	Average Daily Prenatal Melon, Berry, and Citrus Fruit Intake					
	Model 1			Model 2		
Variable	*β*	p-value	95% CI	*β*	p-value	95% CI
Convenience-store distance	0.06	0.20	[−0.03, 0.14]	0.05	0.33	[−0.05, 0.14]
Fast-food restaurant distance	0.00	0.96	[−0.12, 0.12]	0.01	0.90	[−0.12, 0.14]
Grocery-store distance	−0.03	0.60	[−0.15, 0.09]	−0.04	0.50	[−0.17, 0.08]
Convenience-store density	0.07	0.33	[−0.07, 0.20]	0.06	0.35	[−0.07, 0.20]
Fast-food restaurant density	−0.05	0.38	[−0.18, 0.07]	−0.05	0.39	[−0.18, 0.07]
Grocery-store density	0.01	0.93	[−0.24, 0.26]	−0.01	0.96	[−0.26, 0.24]
Infant sex	-	-	-	0.05	0.53	[−0.11, 0.20]
Alcohol use	-	-	-	0.07	0.66	[−0.24, 0.38]

***Note:** Model 1: F(6, 80) = 0.59, p = 0.737, R^2 = 0.04. Model 2: F(8, 76.1) = 0.52, p = 0.839.

Table S2. Multivariate linear regression models predicting average daily prenatal dark, leafy green and red–orange vegetable intake in association with built food environment proximity and potential confounders.*

	Average Daily Prenatal Dark, Leafy Green and Red–Orange Vegetable Intake					
	Model 1			Model 2		
Variable	*β*	p-value	95% CI	*β*	p-value	95% CI
Convenience-store distance	−0.01	0.54	[−0.05, 0.03]	−0.01	0.48	[−0.06, 0.03]
Fast-food restaurant distance	−0.02	0.47	[−0.07, 0.03]	−0.02	0.49	[−0.08, 0.04]
Grocery-store distance	0.02	0.56	[−0.04, 0.07]	0.01	0.60	[−0.04, 0.07]
Convenience-store density	−0.03	0.37	[−0.09, 0.03]	−0.03	0.39	[−0.09, 0.03]
Fast-food restaurant density	0.00	0.88	[−0.05, 0.06]	0.00	0.97	[−0.05, 0.06]
Grocery-store density	0.04	0.52	[−0.07, 0.15]	0.03	0.62	[−0.08, 0.14]
Infant sex	-	-	-	0.00	0.92	[−0.07, 0.07]
Alcohol use	-	-	-	0.05	0.49	[−0.09, 0.19]

***Note:** Model 1: F(6, 80) = 0.37, p = 0.895, R^2 = 0.03. Model 2: F(8, 76.1) = 0.34, p = 0.949.

Table S3. Multivariate linear regression models predicting average daily prenatal nut and seed intake in association with built food environment proximity and potential confounders.*

	Average Daily Prenatal Nut and Seed Intake					
	Model 1			Model 2		
Variable	*β*	p-value	95% CI	*β*	p-value	95% CI
Convenience-store distance	0.03	0.23	[−0.02, 0.07]	0.02	0.34	[−0.02, 0.07]
Fast-food restaurant distance	−0.02	0.47	[−0.08, 0.04]	−0.01	0.70	[−0.08, 0.05]
Grocery-store distance	0.00	0.97	[−0.06, 0.06]	−0.01	0.84	[−0.07, 0.06]
Convenience-store density	0.01	0.74	[−0.06, 0.08]	0.01	0.81	[−0.06, 0.08]
Fast-food restaurant density	0.02	0.46	[−0.04, 0.08]	0.03	0.40	[−0.04, 0.09]
Grocery-store density	0.05	0.42	[−0.07, 0.18]	0.05	0.47	[−0.08, 0.18]
Infant sex	-	-	-	0.03	0.41	[−0.05, 0.11]
Alcohol use	-	-	-	−0.02	0.82	[−0.18, 0.14]

***Note:** Model 1: F(6, 80) = 0.94, p = 0.470, R^2 = 0.07. Model 2: F(8, 76.1) = 0.78, p = 0.618.

Table S4. Multivariate linear regression models predicting average daily prenatal intake of fish high in omega-3 in association with built food environment proximity and potential confounders.*

	Average Daily Prenatal Intake of Fish high in Omega-3					
	Model 1			Model 2		
Variable	*β*	p-value	95% CI	*β*	p-value	95% CI
Convenience-store distance	0.00	0.12	[0.00, 0.01]	0.00	0.22	[−0.00, 0.01]
Fast-food restaurant distance	0.00	0.85	[−0.01, 0.01]	0.00	0.82	[−0.01, 0.01]
Grocery-store distance	0.00	0.40	[0.00, 0.01]	0.00	0.45	[−0.01, 0.01]
Convenience-store density	0.00	0.67	[−0.01, 0.01]	0.00	0.62	[−0.01, 0.01]
Fast-food restaurant density	0.00	0.26	[0.00, 0.01]	0.00	0.39	[−0.00, 0.01]
Grocery-store density	0.01	0.30	[−0.01, 0.02]	0.01	0.47	[−0.01, 0.02]
Infant sex	-	-	-	0.00	0.81	[−0.01, 0.01]
Alcohol use	-	-	-	0.02	0.12	[−0.01, 0.04]

*__Note:__ Model 1: F(6, 80) = 1.07, p = 0.387, R^2 = 0.07. Model 2: F(8, 76.1) = 1.15, p = 0.342.
